# Horsley’s Antiseptic Wax

**Published:** 1897-05

**Authors:** 


### HORSLEY'S ANTISEPTIC WAX.

The value of Horsley's antiseptic wax for controlling undue bleeding from the cut surface of a bone can not be over estimated. This wax is easily prepared, is antiseptic, and is always ready for use. It consists of beeswax, seven parts; almond oil, one part; and salicylic acid, one part. Several years ago Professor Keen introduced this preparation into the Jefferson Hospital. He uses it extensively to arrest bleeding from the bone during an operation. The bleeding that sometimes follows the sawing of a bone during an amputation, excision, or resection can be checked by pressing a small quantity of the wax against the bleeding surface. After the removal of a growth from the jaw, such as an exostosis or an epulis, the bleeding can be readily controlled by an application of this agent. In bleeding from diploe or cancellous bone this wax is of great service.—*College and Clinical Record.*

